# Supplementary material for: Knowledge, attitude and practice of hepatitis B infection prevention among nursing students in the Upper West Region of Ghana: A cross-sectional study
Source: PLoS One. 2021 Oct 14;16(10):e0258757. doi: 10.1371/journal.pone.0258757 (PMC8516292; doi:10.1371/journal.pone.0258757)
Supplement: S1 Table — (PDF) [file pone.0258757.s001.pdf]

KNOWLEDGE, ATTITUDE AND PRACTICE (KAP) SCORING SCALE

**Section B: Knowledge Item questions**		
**Question**	**Option checked [✓]**	**Score (0 or 1)**
11. Is Hepatitis B infection caused by a partial double stranded DNA virus?	Yes [✓]<br>No [✓]<br>Not sure [✓]	1<br>0<br>0
12. Is Jaundice a symptom of hepatitis B infection?	Yes [✓]<br>No [✓]<br>Not sure [✓]	1<br>0<br>0
13. Do all hepatitis B infected people present with signs/symptoms?	Yes [✓]<br>No [✓]<br>Not sure [✓]	0<br>1<br>0
14. Can carriers of hepatitis B who are not sick pass the infection to others?	Yes [✓]<br>No [✓]<br>Not sure [✓]	1<br>0<br>0
15. Is hepatitis B virus transmitted via the faeco-oral route?	Yes [✓]<br>No [✓]<br>Not sure [✓]	0<br>1<br>0
16. Is hepatitis B infection transmitted through casual contact such as holding of hands?	Yes [✓]<br>No [✓]<br>Not sure [✓]	0<br>1<br>0
17. Can hepatitis B infection be transmitted through contaminated blood and blood products?	Yes [✓]<br>No [✓]<br>Not sure [✓]	1<br>0<br>0
18. Can hepatitis B infection be transmitted by un-sterilized syringes, needles and surgical instruments?	Yes [✓]<br>No [✓]<br>Not sure [✓]	1<br>0<br>0
19. Can hepatitis B infection be transmitted via unprotected sex?	Yes [✓]<br>No [✓]<br>Not sure [✓]	1<br>0<br>0
20. Can hepatitis B infection be passed from a mother to her baby at birth?	Yes [✓]<br>No [✓]<br>Not sure [✓]	1<br>0<br>0
21. Is hepatitis B infection diagnosed by serological Rapid Diagnostic Test?	Yes [✓]<br>No [✓]<br>Not sure [✓]	1<br>0<br>0
22. Can diagnosis of hepatitis B infection be done by a molecular test?	Yes [✓]<br>No [✓]<br>Not sure [✓]	1<br>0<br>0
23. Is hepatitis B infection curable?	Yes [✓]<br>No [✓]<br>Not sure [✓]	0<br>1<br>0

24. Can hepatitis B virus cause liver cancer?	Yes [✓]<br>No [✓]<br>Not sure [✓]	1<br>0<br>0
25. Is the hepatitis B vaccine made from human blood?	Yes [✓]<br>No [✓]<br>Not sure [✓]	0<br>1<br>0
26. Does hepatitis B vaccination prevent Hepatitis B infection?	Yes [✓]<br>No [✓]<br>Not sure [✓]	1<br>0<br>0
27. Does hepatitis B vaccine protect against liver cancer?	Yes [✓]<br>No [✓]<br>Not sure [✓]	1<br>0<br>0
28. Does hepatitis B infection have post-exposure prophylaxis?	Yes [✓]<br>No [✓]<br>Not sure [✓]	1<br>0<br>0
**Section C: Attitude Item Questions**		
**Question**	**Option checked [✓]**	**Score (0 or 1)**
29. You are at risk of getting hepatitis B infection	Strongly Agree [✓]<br>Agree [✓]<br>Uncertain [✓]<br>Disagree [✓]<br>Strongly disagree [✓]	1<br>1<br>0<br>0<br>0
30. Occasional contact with blood will not necessarily increase my risk of getting hepatitis B infection	Strongly Agree [✓]<br>Agree [✓]<br>Uncertain [✓]<br>Disagree [✓]<br>Strongly disagree [✓]	0<br>0<br>0<br>1<br>1
31. Wearing personal protective equipment during surgery is unnecessary	Strongly Agree [✓]<br>Agree [✓]<br>Uncertain [✓]<br>Disagree [✓]<br>Strongly disagree [✓]	0<br>0<br>0<br>1<br>1
32. Hepatitis B vaccination is unnecessary because acquiring hepatitis B infection is not as serious as HIV infection	Strongly Agree [✓]<br>Agree [✓]<br>Uncertain [✓]<br>Disagree [✓]<br>Strongly disagree [✓]	0<br>0<br>0<br>1<br>1
33. Hepatitis B infection is not potentially serious because people who acquire it live normal lives	Strongly Agree [✓]<br>Agree [✓]<br>Uncertain [✓]<br>Disagree [✓]<br>Strongly disagree [✓]	0<br>0<br>0<br>1<br>1

34. Hepatitis B infection is not potentially serious because it is treatable	Strongly Agree [✓]<br>Agree [✓]<br>Uncertain [✓]<br>Disagree [✓]<br>Strongly disagree [✓]	0<br>0<br>0<br>1<br>1
35. Occasional needle pricks don't require reporting to the healthcare authorities	Strongly Agree [✓]<br>Agree [✓]<br>Uncertain [✓]<br>Disagree [✓]<br>Strongly disagree [✓]	0<br>0<br>0<br>1<br>1
36. Occasional blood or body fluid splashes on the face don't require any reporting.	Strongly Agree [✓]<br>Agree [✓]<br>Uncertain [✓]<br>Disagree [✓]<br>Strongly disagree [✓]	0<br>0<br>0<br>1<br>1
**Section D: Practice Item Questions**		
**Question**	**Option checked [✓]**	**Score (0 or 1)**
37. Have you screened for Hepatitis B infection?	Yes [✓]<br>No [✓]	1<br>0
38. Have you ever taken the hepatitis B vaccine?	Yes [✓]<br>No [✓]	1<br>0
39. If yes to Q 38, how many doses of hepatitis B vaccine did you receive?	1 dose [✓]<br>2 doses [✓]<br>3 doses [✓]<br>> 3 doses [✓]	0<br>0<br>1<br>1
40. Have you done a post hepatitis B vaccination antibody test?	Yes [✓]<br>No [✓]	1<br>0
41. Do you change gloves for each patient during blood collection?	Always [✓]<br>Sometimes [✓]<br>Never [✓]	1<br>0<br>0
42. Do you recap needles after use?	Always [✓]<br>Sometimes [✓]<br>Never [✓]	0<br>0<br>1
43. To the best of your knowledge have you acquired needle stick injuries in the past?	Always [✓]<br>Sometimes [✓]<br>Never [✓]	0<br>0<br>1
44. To the best of your knowledge have you splashed blood/body fluids on your body?	Always [✓]<br>Sometimes [✓]<br>Never [✓]	0<br>0<br>1
