# Supplementary material for: Knowledge, attitude and practice of hepatitis B infection prevention among nursing students in the Upper West Region of Ghana: A cross-sectional study
Source: PLoS One. 2021 Oct 14;16(10):e0258757. doi: 10.1371/journal.pone.0258757 (PMC8516292; doi:10.1371/journal.pone.0258757)
Supplement: S1 Questionnaire — (PDF) [file pone.0258757.s002.pdf]

UNIVERSITY FOR DEVELOPMENT STUDIES, TAMALE

SCHOOL OF ALLIED HEALTH SCIENCES

DEPARTMENT OF PUBLIC HEALTH

QUESTIONNAIRE FOR KAP OF HEPATITIS B INFECTION PREVENTION SURVEY

I am an MPhil student pursuing a programme in Community Health and Development, University for Development Studies, Tamale. This questionnaire is designed to assess the knowledge, attitude and practices of hepatitis B infection prevention among nursing students in the Upper West Region. Kindly take time to read and answer each question carefully by ticking **[✓]** the best alternative that represents your response. The findings of this study would be useful in the improvement of the knowledge and attitude of nursing students in the region, which is expected to culminate in improved practices of hepatitis B infection prevention among nursing students and healthcare workers in general. You are assured of utmost confidentiality concerning the information you would give out. Therefore, you are advised not to write your name. It would take about 10 minutes to complete the form. Consenting to participate in this survey is purely voluntary. Thank you for your participation!!!

**Section A: Demographics || Please for each question tick only one response option**	
1. What is your sex?	Male [ ] Female [ ]
2. How old were you at your last birthday?	[ ] years
3. What is your marital status?	Married [ ] Consensual union [ ] Separated [ ]<br>Divorced [ ] Widowed [ ] Never married [ ]
4. What is your religious affiliation?	No religion [ ] Catholic [ ] Protestant [ ]<br>Pentecostal/Charismatic [ ] Other Christian [ ]<br>Islam [ ] Traditionalist [ ]<br>Others [ ] Specify______________________

5. What ethnic group do you belong to?	Dagaaba/ Waala/ Sisaala [ ]<br>Frafra/ Kasena/ Builsa [ ]<br>Dagomba/ Gonja/ Mamprusi [ ] Akan [ ]<br>Ga/Dangme [ ] Ewe [ ]<br>Others [ ] Specify __________________
6. What is your permanent residential location?	Rural [ ] Urban [ ]
7. What is the name of your college?	Wa NTC [ ] Lawra NTC [ ]
8. What programme of study are you pursuing?	RGN [ ] NAC [ ]
9. What is your level of study?	1st year [ ] 2nd year [ ] 3rd year [ ]
10. What is your parent/guardian's level of formal education?	None [ ] Primary [ ] Middle school/JSS [ ]<br>SSS [ ] Tertiary [ ]
**Section B: Knowledge Item questions || Please for each question, tick only one response option**	
11. Is Hepatitis B infection caused by a partial double stranded DNA virus?	Yes [ ] No [ ] Not sure [ ]
12. Is Jaundice a symptom of hepatitis B infection?	Yes [ ] No [ ] Not sure [ ]
13. Do all hepatitis B infected persons present with symptoms?	Yes [ ] No [ ] Not sure [ ]
14. Can carriers of hepatitis B who are not sick pass the infection to others?	Yes [ ] No [ ] Not sure [ ]
15. Is hepatitis B virus transmitted via the faeco-oral route?	Yes [ ] No [ ] Not sure [ ]
16. Is hepatitis B infection transmitted through casual contact such as holding of hands?	Yes [ ] No [ ] Not sure [ ]
17. Can hepatitis B infection be transmitted by contaminated blood and blood products?	Yes [ ] No [ ] Not sure [ ]
18. Can hepatitis B infection be transmitted by un-sterilized syringes, needles and surgical instruments?	Yes [ ] No [ ] Not sure [ ]
19. Can hepatitis B infection be transmitted via unprotected sex?	Yes [ ] No [ ] Not sure [ ]

20. Can hepatitis B infection be passed from a mother to her baby at birth?	Yes [ ] No [ ] Not sure [ ]
21. Is hepatitis B infection diagnosed by serological Rapid Diagnostic Test?	Yes [ ] No [ ] Not sure [ ]
22. Can diagnosis of hepatitis B infection be done by a molecular test?	Yes [ ] No [ ] Not sure [ ]
23. Is hepatitis B infection curable?	Yes [ ] No [ ] Not sure [ ]
24. Can hepatitis B virus cause liver cancer?	Yes [ ] No [ ] Not sure [ ]
25. Is the hepatitis B vaccine made from human blood?	Yes [ ] No [ ] Not sure [ ]
26. Does hepatitis B vaccination prevent hepatitis B infection?	Yes [ ] No [ ] Not sure [ ]
27. Does the hepatitis B vaccine protect against liver cancer?	Yes [ ] No [ ] Not sure [ ]
28. Does hepatitis B infection have post-exposure prophylaxis?	Yes [ ] No [ ] Not sure [ ]
**Section C: Attitude Item Questions || Please for each statement, tick only one response option**	
29. You are at risk of getting hepatitis B infection	Strongly agree [ ] Agree [ ] Uncertain [ ] Disagree [ ] Strongly disagree [ ]
30. Occasional contact with blood will not necessarily increase my risk of getting hepatitis B infection	Strongly agree [ ] Agree [ ] Uncertain [ ] Disagree [ ] Strongly disagree [ ]
31. Wearing personal protective equipment during surgery is unnecessary	Strongly agree [ ] Agree [ ] Uncertain [ ] Disagree [ ] Strongly disagree [ ]
32. Hepatitis B vaccination is unnecessary because acquiring hepatitis B infection is not as serious as HIV infection	Strongly agree [ ] Agree [ ] Uncertain [ ] Disagree [ ] Strongly disagree [ ]
33. Hepatitis B infection is not potentially serious because people who acquire it live normal lives	Strongly agree [ ] Agree [ ] Uncertain [ ] Disagree [ ] Strongly disagree [ ]
34. Hepatitis B infection is not potentially serious because it is treatable	Strongly agree [ ] Agree [ ] Uncertain [ ] Disagree [ ] Strongly disagree [ ]

35. Occasional needle pricks don't require reporting to the healthcare authorities	Strongly agree [ ] Agree [ ] Uncertain [ ] Disagree [ ] Strongly disagree [ ]
36. Occasional blood or body fluid splashes on the face don't require any reporting.	Strongly agree [ ] Agree [ ] Uncertain [ ] Disagree [ ] Strongly disagree [ ]
**Section D: Practice Item Questions || Please for each question, tick only one response option**	
37. Have you screened for Hepatitis B infection?	Yes [ ] No [ ]
38. Have you ever taken the hepatitis B vaccine?	Yes [ ] No [ ]
39. If yes to Q 38, how many doses of hepatitis B vaccine did you receive?	1 dose [ ] 2 doses [ ] 3 doses [ ] > 3 doses [ ]
40. Have you done a post hepatitis B vaccination antibody test?	Yes [ ] No [ ]
41. Do you change gloves for each patient during blood collection?	Always [ ] Sometimes [ ] Never [ ]
42. Do you recap needles after use?	Always [ ] Sometimes [ ] Never [ ]
43. To the best of your knowledge have you acquired needle stick injuries in the past?	Always [ ] Sometimes [ ] Never [ ]
44. To the best of your knowledge have you splashed blood/body fluids on your body?	Always [ ] Sometimes [ ] Never [ ]
**Section E: Reasons for non-uptake of Hepatitis B vaccine**<br>**If your answer is NO to question 38, why have you not received the hepatitis B vaccine? Please for each question, tick one option only. If YES to question 38, please do not answer questions 45-50**	
45. I do not know where to go and receive it	Yes [ ] No [ ]
46. I am not at risk of infection	Yes [ ] No [ ]
47. I think the vaccine is expensive	Yes [ ] No [ ]
48. I am afraid of the side effects of the vaccine	Yes [ ] No [ ]
49. The vaccine is not effective	Yes [ ] No [ ]
50. I had hepatitis B infection in the past	Yes [ ] No [ ]

END OF SURVEY. THANK YOU ONCE AGAIN FOR YOUR PARTICIPATION!!!
