# Supplementary material for: Knowledge, attitude and practice of hepatitis B infection prevention among nursing students in the Upper West Region of Ghana: A cross-sectional study
Source: PLoS One. 2021 Oct 14;16(10):e0258757. doi: 10.1371/journal.pone.0258757 (PMC8516292; doi:10.1371/journal.pone.0258757)
Supplement: Attachment — Submitted filename: Response to reviewers.docx [file pone.0258757.s005.docx]

**POINT-BY-POINT RESPONSE TO REVIEWERS’ COMMENTS**

**Ref:** Submission ID [PONE-D-21-21003] - [EMID:4da1bd33a6fc8347]

**Title:** Knowledge, attitude and practice of hepatitis B infection prevention among nursing students in the Upper West Region of Ghana: a cross-sectional study

**Editorial comments**

**General response**

We are very grateful to the editor for the positive feedback regarding our manuscript. We are also thankful for the editorial comments which we have addressed. We hope that our manuscript now meets the editorial requirements of the journal.

**Comment 1**

Please ensure that your manuscript meets PLOS ONE’s style requirements, including those for file naming.

**Response 1**

Thank you for this reminder. We have referred to PLOS One’s style requirements as exemplified by the PLOS One templates for title page and manuscript body formatting guidelines. We have also named our files according to the requirements of the journal.

**Comment 2**

Please include additional information regarding the survey or questionnaire used in the study and ensure that you have provided sufficient details that others could replicate the analyses.

**Response 2**

We are grateful for this guideline. We have included more information on the survey and the questionnaire as found under “data collection” of the methods section. We have also added the questionnaire as a supplementary information file.

**Comment 3**

Please state whether you validated the questionnaire prior to testing on study participants. Please provide details regarding the validation group within the methods section.

**Response 3**

Thank you for this comment. The questionnaire was validated before data collection. Details of the validation group and the validation process can now be found in the methods section under a fresh sub-heading “validity and reliability of the instrument”.

**Comment 4**

In your Data Availability statement, you have not specified where the minimum data set underlying the results described in your manuscript can be found. Upon re-submitting your revised manuscript, please upload your study’s minimal underlying data set as either Supporting Information files or to a stable, public repository and include the relevant URLs, DOIs, or accession numbers within your revised cover letter. We will update your Data Availability statement to reflect the information you provide in your cover letter.

**Response 4**

We are grateful for your guidelines. We have now uploaded the minimum underlying data set of our study as a supporting information file.

**Comment 5**

Your ethics statement should only appear in the Methods section of your manuscript. If your ethics statement is written in any section besides the methods, please move it to the methods section and delete it from any other section.

**Response 5**

As required, we have moved the ethics statement of this study to the methods section. Consequently, we have deleted it from its previous position on the original manuscript.

**Reviewers’ comments**

**Reviewer 1**

**Comment 1**

The analysis performed for regression is very vague and the results are not consistent with the objectives. It is also difficult for the reader to understand (Table 6).

**Response 1**

We thank the reviewer for the comment. We have revised the wording of some parts of the results to improve upon clarity of the interpretation of the results from the regression model presented in Table 6. We think that the regression analysis we performed is not inconsistent with our study objectives. The last objective of our study was to assess the factors associated with the practice of hepatitis B infection prevention. The dependent variable “practice of Hepatitis B infection prevention” had three main outcomes which were ordered in nature. The multivariate generalised logistic regression analysis we performed served to assess the association between practice of hepatitis B infection prevention as the dependent variable, and knowledge of hepatitis B infection prevention, attitude towards hepatitis B infection prevention and the socio-demographic characteristics of the respondents as the independent variables

Table 6 displays the results of the multivariate generalised ordered logistic regression model we run. Although the result was from the running of a single model, it generated two outputs in accordance with the partial parallel regression/ partial proportional odds assumption. We have clearly explained this under the data analysis part of the methods section.

To further improve upon the clarity of the results in Table 6, we have revised our interpretations of the results in the main manuscript.

**Comment 2**

Multivariate logistic regression was not performed.

**Response 2**

We thank the reviewer for this comment. Perhaps our wording of some parts of the methods section that was not clear to the reviewer but we indeed performed multivariate logistic regression. Based on our understanding of multivariate logistic regression, we think that the generalised ordered logistic regression modelling that we performed is a form of multivariate logistic regression. As indicated above, our dependent variable, practice of hepatitis B infection prevention is a polychotomous ordered categorical variable (poor, moderate and good practice of hepatitis B infection prevention). Therefore, initially a multivariate ordered logistic regression was performed by regressing practice of hepatitis B infection on knowledge of hepatitis B infection, attitude towards hepatitis B infection and the socio-demographic characteristics of the respondents. As explained under data analysis of methods section, the multivariate ordered logistic regression model failed the Brant test of proportional odds assumption. A multivariate generalised ordered logistic regression model with autofit option was the best alternative as cited by the relevant literature under methods section. In sum, the regression modelling that we performed was multivariate.

**Comment 3**

The model assumptions in the materials and methods section should be written in full and it should be noted how this analysis was performed (more details).

**Response 3**

Thank you for your comment. As rightly pointed out by the reviewer, we have clearly and fully explained the parallel regression/ proportional assumption as well as the partial proportional odds assumptions under data analysis of the methods section. We have also indicated in detail under data analysis, how the analysis was performed. In fact, the entire analysis subsection of the methods has been rewritten to improve upon clarity of our methodological descriptions.

**Comment 4**

Is there a significant difference in the study of people (demographic variables) in age group, gender, marital status and place of residence, etc. (Table 1)? Because these variables are confusing, their differences can affect multivariate regression analysis.

**Response 4**

We are grateful for your comment. Table 1 displays a descriptive analysis of the socio-demographic characteristics of the respondents in frequencies and percentages. The analysis presented in Table 1 was univariate for the purpose of only describing the proportions of respondents under each category of each variable. We did not conduct bivariate analysis to show differences between socio-demographic variables and any dependent variable (i.e practice of hepatitis B infection). We explored such significant associations (differences) in the multivariate regression modelling. As indicated by Table 6, the socio-demographic characteristics of the respondents which showed statistically significant associations when regressed on practice of hepatitis B infection prevention in our multivariate generalised logistic regression model were; sex, marital status, level of education and parent’s level of education. We described these significant associations of the socio-demographic characteristics with the practice of hepatitis B in the results section and further discussed these findings under the discussion section**.**

**Reviewer 2**

**Comment 1**

It is advised to take the assistance of native speakers or editing services to improve the quality of writing. I could see many fundamental write-up-related issues like correct grammar and tense, spell out of numbers below ten, complete form in the first use of abbreviations, and inappropriate syntax, etc. Even if authors can’t afford the editing services, they are advised to use Grammarly premium to omit such basics.

**Response 1**

We thank the reviewer these valuable comments. We have edited and proof read our manuscript with Grammarly premium.

**Comment 2**

Additionally, write is up is too much lengthy. The introduction must be concise, justifying the study, and usually of three average paragraphs. But, the study is almost three pages. So, please omit too much unimportant information (waste words) and findings of other studies in detail. However, detailing the method is expected and justified!

**Response 2**

Thank you for your comment. We agree with you that the write-up, especially the introduction was lengthy. We have therefore identified and deleted some irrelevant statements under the introduction section.

**Comment 3**

Topic: The study participants are being taken from only two colleges from a specific region of the country. So, generalization for the whole nation may not be appropriate and relevant. Therefore, it would be better not to use the term “in Ghana” in the topic.

**Response 3**

We agree with the reviewer’s comment. We have rephrased the title as “Knowledge, attitude and practice of hepatitis B infection prevention among nursing students in the Upper West Region of Ghana: a cross-sectional study. We have specified the region from which the study was conducted.

**Comment 4**

Introduction: It is too lengthy to go through. Even though there are no word constraints in journal guidelines, it is better to concise and condense the introduction. Just use the required information only.

**Response 4**

We agree with the reviewer that the write-up, especially the introduction section was lengthy. We have therefore identified and deleted some less important statements under the introduction section.

**Comment 5**

**Results:**

There is a difference in the percentage of ‘never married’ participants in tables and descriptions in words.

**Response 5**

Thank you for your comment. We have corrected the error in reporting the proportion of “never married” respondents. The correct figure is 90.8 %.

**Comment 6**

It is described that “…the nursing students recognized MTCT (79.8%), unprotected sex (81.1%) and unsterilized surgical instruments (85.6%) as the major route of transmission of hepatitis B infection.” And, what about contaminated blood/products? Again, 90.8% have recognized it as the significant route!!

**Response 6**

We have now reported the proportion of respondents who recognised contaminated blood/ products as a transmission route for hepatitis B infection as 90.8%.

**Comment 7**

The term “48.5% knew hepatitis B vaccine as an anticancer vaccine” sounds unusual. So you had better write 48.5% of them knew the hepatitis B vaccine protects against liver cancer.

**Response 7**

We are grateful for this comment. We have re-written the statement to read “48.5% of them knew that hepatitis B vaccine protects against liver cancer”.

**Comment 8**

While describing any finding, it is better to use the parameter with a higher value first, followed by lower values.

**Response 8**

We have now reported higher values of parameters of the results before the lower values.

**Comment 9**

**Statistics:**

In table 5, both mean (SD) and median (IQR) for composite scores are kept without any statistical consideration. Generally, we use mean and standard deviation for normal data, and for non-normally distributed data, we use the median (IQR) for continuous variables. Would you please mention clearly in method as well regarding distribution of data and appropriate use of statistical tests?

**Response 9**

We are grateful for comment. The composite scores for knowledge, attitude and practice of hepatitis B infection prevention were not normally distributed. Therefore, although several summary statistics have been reported for KAP of hepatitis B infection prevention, composite median scores with interquartile ranges (IQRs) have now been used as the measure of central tendency for assessing KAP of hepatitis B infection prevention. We have therefore replaced the mean and standard deviation values with the median and IQR values in the abstract and in the description of the results in the main manuscript. Additionally, the distribution as well as appropriate statistical tests of the data for KAP of hepatitis B infection prevention have now been stated clearly under data analysis of the methods section. Consequently, we have included skewness and kurtosis as measures of data distribution under both the methods and results sections.

**Comment 10**

**Discussion:**

Comparison and contrast with other studies have been made extensively. Therefore, please add “take away message” from your research to the existing medical literature.

**Response 10**

We thank reviewer for the comment. We think that we have already presented “take away messages in the form of practical policy and research implications in the discussions of the results. The last part of each paragraph in the discussion section sought to bring out the implications of our findings in the form of take-away messages. However, in addition to what we have already presented we have included “Therefore, management of nursing training colleges should promote education on hepatitis B infection prevention as well as enforce proper professional conduct among the nursing students” as a policy implication under the discussion for the overall poor practice of the nursing students. We have also included “However, we propose that further studies be conducted to explore the detailed role marriage plays in promoting good practice of hepatitis B infection prevention” as an implication for further studies.

**Reviewer 3**

**Comment 1**

It is well written article, with a very good sample size. Here are my comments, hope these will help in improvement of manuscript and quality of information.

**Response 1**

We are very grateful to the reviewer for appreciating our work. We are also thankful for your constructive comments offered to help improve the quality of our work.

**Comment 2**

Abstract: Overall abstract do not represent the study presented but need correction. Editing of English is advised as using a passive voice instead of active voice.

**Response 2**

Thank you for your comment. We have revised the abstract as a whole especially the background, results and conclusion of the abstract. To the best of our knowledge, we have now used active voice in our write-up.

**Comment 3**

Background: The abstract mentioned the background as “Good knowledge, attitude and practice (KAP) of hepatitis B infection (HBI) prevention are essential to disease prevention" which is not sufficient as a background of the study.

**Response 3**

We have added that “Hepatitis B infection remains a public health threat associated with undesirable statistics of morbidity and mortality”. Therefore, the background of the abstract now reads “Hepatitis B infection remains a public health threat associated with undesirable statistics of morbidity and mortality. Good knowledge, attitude and practice (KAP) of hepatitis B infection (HBI) prevention are essential to hepatitis B infection control. However, there is limited evidence on the KAP of HBI prevention among nursing students, who are significantly exposed to HBI. We assessed KAP of HBI prevention and the factors associated with the practice of HBI prevention”.

**Comment 4**

Methodology: Methodology is not clearly presented in abstract, need correction.

**Response 4**

We are grateful for the comment. The methods section of our manuscript covered the study setting, design, population and sampling, data collection method and period and data analysis including software and version used for data analysis of both the descriptive and inferential statistical analysis performed. Therefore, to the best of our knowledge, we think that the methods section of the abstract exhausted the relevant aspects of a typical methods section of an abstract.

**Comment 5**

Reference: Authors are advised to check the reference for completeness and journal requirements,

**Response 5**

We are grateful for your constructive comment. We have inspected the references and ensured their completeness and conformation with the referencing style recommended by PLOS ONE.
