# Supplementary material for: Knowledge, attitude and practice of hepatitis B infection prevention among nursing students in the Upper West Region of Ghana: A cross-sectional study
Source: PLoS One. 2021 Oct 14;16(10):e0258757. doi: 10.1371/journal.pone.0258757 (PMC8516292; doi:10.1371/journal.pone.0258757)
Supplement: Attachment — Submitted filename: Response to reviewers.docx [file pone.0258757.s006.docx]

**POINT-BY-POINT RESPONSE TO REVIEWERS’ COMMENTS**

**Ref:** Submission ID [PONE-D-21-21003R1] - [EMID:72516b38fa838aa1]

**Title:** Knowledge, attitude and practice of hepatitis B infection prevention among nursing students in the Upper West Region of Ghana: a cross-sectional study

**Editorial comments**

**General response**

We are very grateful to the editor for the positive feedback regarding our manuscript. We hope that this current version of our manuscript will be suitable for publication.

**Journal Requirements:**
Please review your reference list to ensure that it is complete and correct. If you have cited papers that have been retracted, please include the rationale for doing so in the manuscript text, or remove these references and replace them with relevant current references. Any changes to the reference list should be mentioned in the rebuttal letter that accompanies your revised manuscript. If you need to cite a retracted article, indicate the article’s retracted status in the References list and also include a citation and full reference for the retraction notice.

**Response**

We have reviewed and updated our reference list. The following are the modifications made;

**Reference 1**

The page numbers of the first reference has been updated to “xxx-xxx” in the current version of the manuscript. The first reference now reads;

1.	Jacob A, Joy TM, Mohandas S, Lais H, Paul N. Assessment of knowledge, attitude, and practice regarding hepatitis B among medical students in a private medical college in Kochi. Int J Community Med Public Health. 2019;6: xxx–xxx. doi:10.18203/2394-6040.ijcmph20191552

**Reference number 4**

We have updated the list of contributors of the 4^th^ reference in the previous manuscript. We have included “Apica B, Awuku Y, Cunha L, et al.” The reference now reads; Spearman CW, Afihene M, Ally R, Apica B, Awuku Y, Cunha L, et al. Hepatitis B in sub-Saharan Africa: strategies to achieve the 2030 elimination targets. The Lancet Gastroenterology & Hepatology. 2017;2: 900–909. doi:10.1016/S2468-1253(17)30295-9

**Reference number 6**

“Doi” in reference number six was duplicated in the previous version of the manuscript as “doi.doi”. Therefore, we have reviewed it to read; Dongdem JT, Kampo S, Soyiri IN, Asebga PN, Ziem JB, Sagoe K. Prevalence of hepatitis B virus infection among blood donors at the Tamale Teaching Hospital, Ghana (2009). BMC Res Notes. 2012;5: 115. doi:10.1186/1756-0500-5-115

**Reference number 21**

We have deleted “1” depicted wrongly as the page number of the 21^st^ reference in the previous version of the manuscript. The reference now reads; Al-Hussami M. Knowledge and acceptance of hepatitis B vaccine. Internet Journal of Health Care Administration. 2001;2. Available: https://ispub.com/IJHCA/2/1/5725

**Reference numbers 23, 24 and 25**

Reference numbers 23, 24 and 25 in the previous manuscript have been correctly ordered in the newly revised manuscript. These references are arranged in the previous manuscript as follows;

23. Daud KAM, Khidzir NZ, Ismail AR, Abdullah FA. Validity and reliability of instrument to measure social media skills among small and medium entrepreneurs at Pengkalan Datu River. 2018;7: 1026–1037.

24. Cronbach LT. Coefficient alpha and the internal structure of tests. Psychometrika. 1951;16: 297–334. doi:10.1007/BF02310555

25. Creswell JW, Creswell JD. Research Design: Qualitative, Quantitative, and Mixed Methods Approach. 5th ed. Los Angeles: Sage Publications, Inc.; 2018.

However, after our update the references were re-organised as follows;

23. Creswell JW, Creswell JD. Research Design: Qualitative, Quantitative, and Mixed Methods Approach. 5th ed. Los Angeles: Sage Publications, Inc.; 2018.

24. Daud KAM, Khidzir NZ, Ismail AR, Abdullah FA. Validity and reliability of instrument to measure social media skills among small and medium entrepreneurs at Pengkalan Datu River. 2018;7: 1026–1037.

25. Cronbach LT. Coefficient alpha and the internal structure of tests. Psychometrika. 1951;16: 297–334. doi:10.1007/BF02310555

**Reference number 28**

The page numbers of reference number 28 which was written as 1171-78 has now been fully written. It therefore now reads;

Brant R. Assessing proportionality in the proportional odds model for ordinal logistic regression. Biometrics. 1990;46: 1171–1178. doi:10.2307/2532457

**Reference numbered 31**

After our review, we realised that the 31^st^ reference on the previous version of the manuscript had previously been deleted and therefore not in the manuscript text. Consequently, we have deleted it from the current version. However, the remaining references have been reorganised as their positions have been changed due to the deletion.

**Reference 36**

Reference 36 in the previous version of the manuscript has been updated to a later edition of the book chapter. Therefore, the 36^th^ reference previously listed as

“Prochaska JO, Redding C, Evers K. The transtheoretical model and stage of change. Health behavior and health education Theory, Research, and Practice. 2008. pp. 99–120. Available: https://soh.iums.ac.ir/uploads/beeduhe_0787996149.pdf#page=135” has been updated to;

Prochaska JO, Redding C, Evers K. The transtheoretical model and stages of change. 5th ed. In: K Glanz, B K Rimer, & K “V” Viswanath, editors Health behavior: Theory, research, and practice. 5th ed. Jossey-Bass/Wiley; 2015. pp. 125–148.”

**Reference 38**

We added “In:” to the 38^th^ reference on the previous version of the manuscript, now 37^th^ reference in the revised manuscript. We also replaced “(Eds)” with “editors”. The page number were also written in full as “165-184” and not “165-84”. The reference now reads;

Baranowski T, Perry CL, Parcel GS. How individuals, environments, and health behavior interact: Social cognitive theory. 3rd ed. In: K Glanz, BK, Rimer & FM Lewis, editors Health Behavior and health education: Theory, research, and practice. 3rd ed. San Francisco: Jossey-Bass; 2002. pp. 165–184.

**Reference number 40**

Reference number 40 of the previous version of the manuscript, now reference number 39 of the current version, has been updated from

“Naidoo J, Wills J. Foundations of Health Promotion. Oxford: Baillière Tindall; 2009. Available: http://pdfpremiumfree.com/download/naidoo-and-wills-2009-foundations-of-health-promotion-pdf-pdf/” to;

“Naidoo J, Wills J. Health Promotion: Foundations for Practice. 3rd ed. London: Baillière Tindall; 2009.”

**Reference number 44**

The page numbers of reference number 44 of the previous version of the manuscript, now number 43 on the current version, has been written in full as “109-113” instead of “109-13”. The reference now reads;

Paudel D, Prajapati S, Paneru D. Preventive practices against Hepatitis B: A cross-sectional study among nursing students of Kathmandu, Nepal. J Sci Soc. 2012;39: 109–113. doi:10.4103/0974-5009.105911

**Reviewers' comments:**
**General response**

We are very grateful to the reviewers for the positive feedback concerning our manuscript. We are particularly very excited that both reviewers have acknowledged that we have addressed all comments.

**Reviewer #1**:

Please have the data analyzed by a statistician or epidemiologist and the results reviewed. There is still ambiguity in data analysis. Materials and methods are also confusing.

**Response**

We thank the reviewer for acknowledging that we have addressed all comments raised in our previous submission. Although, the reviewer suggested that our data be re-analysed by a statistician or epidemiologist, no specific issues were raised.

Nevertheless, the authors have background knowledge in statistics and epidemiology as well as regression modelling. We have already cited William (2006 & 2016), an authority in the analysis and interpretation of data involving ordered categorical outcome variables. Using STATA, William (2006 & 2016) explained the methods for such analysis and the interpretation of the data output. We are therefore convinced that our analysis and interpretation of results is appropriate.

The methods and material section covers the KAP-O framework underpinning the study, the study setting, approach and design, sampling, data collection, validity and reliability of study instrument, ethical considerations as well as data analysis. We believe therefore that we have adequately explained the methods we used in our study. We also agree with the unanimous comment by both reviewers that our manuscript has been presented in an intelligible fashion and written in standard English. Therefore, we believe that the methods section as well as the entire manuscript is intelligible enough to be comprehended by the reader.

**Reviewer #2**

Authors have rectified the comments and suggestions in their previous version of the manuscript, so the current version of the manuscript can be considered for the further processing.

**Response**

We are very grateful to the reviewer for this comment. We appreciate the reviewer for commending our work. We are even much happier for the recommendation that our manuscript be considered for further processing.
